# Scalp Necrosis Revealing Severe Giant-Cell Arteritis

**DOI:** 10.1155/2020/8130404

**Published:** 2020-08-14

**Authors:** Safa Idoudi, Marouene Ben Kahla, Fares Mselmi, Badreddine Sriha, A. Guiga, Samia Ayachi, Colandane Belajouza, Mohamed Denguezli

**Affiliations:** ^1^Dermatology Department, Farhat Hached University Hospital, Sousse Medical Faculty, Sousse University, Sousse, Tunisia; ^2^Maxillo Facial Departement, Sahloul Hospital, Sousse Medical Faculty, Sousse University, Sousse, Tunisia; ^3^Anapathology Department, Farhat Hached University Hospital, Sousse Medical Faculty, Sousse University, Sousse, Tunisia; ^4^Internal Medecine Departement, Farhat Hached University Hospital, Sousse Medical Faculty, Sousse University, Sousse, Tunisia

## Abstract

Giant-cell arteritis (GCA), also referred to as temporal arteritis, is the most common primary vasculitis of the elderly involving the extracranial branches of the carotid arteries, in particular, the temporal artery. Patients usually present with temporal headaches, visual impairment, fever, and scalp tenderness. Scalp necrosis associated with GCA is a rare occurrence with approximately 100 cases reported in the literature to date. It is a therapeutic emergency requiring urgent management as it may lead to irreversible loss of vision. To increase awareness of this severe complication, we report a patient with a scalp necrosis revealing a GCA.

## 1. Introduction

Giant-cell arteritis (GCA), also referred to as temporal arteritis, is a common granulomatous vasculitis of medium and large arteries occurring in elderly patients. Common clinical manifestations include intense headache associated to scalp tenderness, jaw claudication, and fatigue. However, some ischemic complications related to severe thrombosis and advanced inflammation may reveal the disease and lead to poor outcomes with high intensity care.

Herein, we report the evaluation and management of a patient with GCA who had severe scalp necrosis.

## 2. Case Report

**Figure 1:**
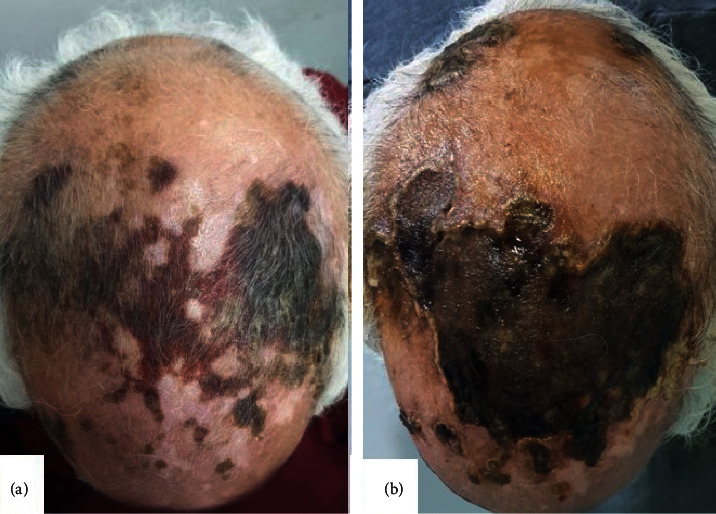
(a) Haemorrhagic patch of the parietal, frontal, and occipital regions of the scalp (day 2). (b) Extensive necrotic ulcerations of the parietal, frontal, and occipital regions of the scalp (day 7).

**Figure 2:**
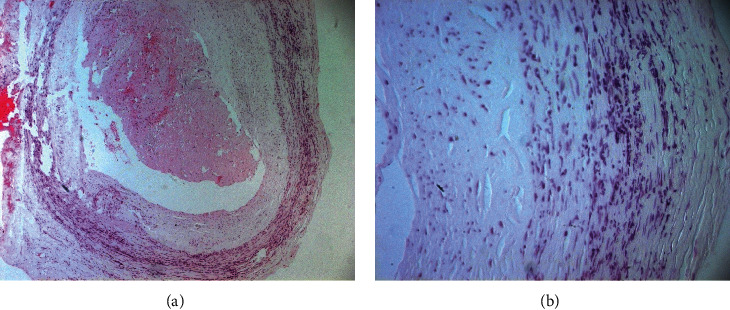
(a) A temporal artery biopsy showing a granulomatous vasculitis of vessel walls. (b) Collections of macrophages, neutrophils, and giant cells in the vessels walls.

A 72-year-old adult presented with a one-week history of painful necrotic ulcerations of the scalp. The patient had a history of hypertension, dyslipidemia, and amputation of the right leg since 8 years for acute ischemia. The lesion started as a haemorrhagic patch of the parietal region of the scalp; then it has subsequently worsened and spread up within 2 days (Figure 1(a)). He reported an acute headache but without visual trouble or polymyalgia rheumatic. Physical examination revealed extensive necrotic ulcerations with obvious hyperesthesia in both parietal and temporal regions of the scalp (Figure 1(b)). Temporal arteries were indurated without pulse on the right side. A clinical diagnosis of scalp necrosis as complication of giant-cell arteritis was suspected, so the patient was admitted to our department and was started on a regimen of oral glucocorticoids 1 mg/kg/day. Ophthalmologic examination did not mention any abnormalities. His erythrocyte sedimentation rate was normal, and his C-reactive protein level was 90 mg per liter. A temporal artery biopsy showed a granulomatous vasculitis of vessel walls with collections of macrophages, neutrophils, and giant cells (Figure 2).

**Figure 3:**
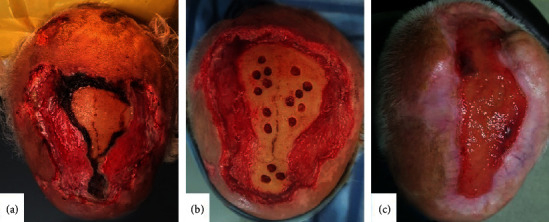
(a) 2 weeks after excision of the necrotic ulcerations performed at the maxillofacial department on day 21. (b) Trepanning followed by multiple negative-pressure wound therapy session (day 120). (c) Partial cicatrisation of the scalp necrosis after one year of follow-up.

Given the clinical appearance and biopsy result, the diagnosis of giant-cell arteritis was approved. Imaging techniques like ultrasound were no more needed though it is of great interest to support the diagnosis. Despite the only onset of oral glucocorticoids with ointment dressings applied twice daily, lesions worsened. Two weeks later, he was referred to maxillofacial surgery department where he underwent during many weeks several surgical operations: excision of the necrotic ulcerations (Figure 3(a)) and trepanning followed by negative-pressure wound therapy (Figure 3(b)). Recovery was uneventful, and there was no further progression of necrosis at 12 months of follow-up. The patient was satisfied with the cosmetic outcome, and cutaneous graft was refused (Figure 3(c)). We did not mention a relapse until the patient died of a myocardial infraction one year later.

## 3. Discussion

**Figure 4:**
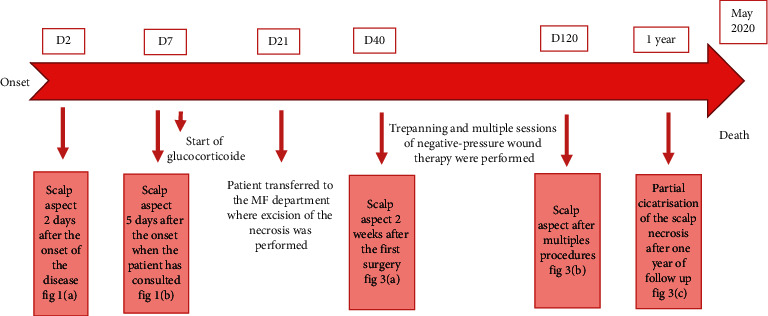
Timeframe figure.

Scalp necrosis is a dramatic but an outstanding complication of GCA. Apart from leading to infection, pain, and prolonged healing wound, scalp necrosis associated with GCA is considered as a prognostic factor for higher mortality and major morbidity [1]. Its onset tends to be insidious over weeks to months and abrupt in up to 20% of patients [2]. It is generally accepted that it is related to not only a relatively advanced giant-cell arteritis [3] but also to a delay in diagnosis and initiation of effective treatment resulting in multivessel occlusion of all four main arteries supplying the temporal scalp [4]. It represents therefore an extreme therapeutic emergency as it can spread in depth resulting in a significant loss of substance with a risk of skull bone necrosis, irreversible visual loss, and severe tongue necrosis [5]. Indeed, despite a richly braided supply of the scalp, ischemia occurs in connection with diffusion of the arteritis phenomenon not only to the scalp vessels but also to the rest of the face with irreversible risk of blindness and lingual necrosis [6], thus encouraging to start high-dose corticosteroid therapy with close monitoring. Recently, Tocilizumab has proven its efficiency especially in severe and rebellious cases like our patient's, but it was difficult to afford it because o its unavailability in our country. Local evolution is most often favorable, but, in the most severe cases, like our patient's, the healing time may exceed one year, with the use of surgical methods (trepanation of the external table, negative-pressure wound therapy, and skin grafting) [7]. (Figure 4)

Despite the early diagnosis of GCA for our patient and close collaboration among specialists, skin necrosis and inflammation continued for a prolonged period, curing after more than year of follow-up which underlines the severity of such complication. Therefore, awareness of GCA initial signs among all health care should be increased.
